# Comparative Transcriptomics Reveals Distinct Patterns of Gene Expression Conservation through Vertebrate Embryogenesis

**DOI:** 10.1093/gbe/evab160

**Published:** 2021-07-10

**Authors:** Megan E Chan, Pranav S Bhamidipati, Heather J Goldsby, Arend Hintze, Hans A Hofmann, Rebecca L Young

**Affiliations:** 1 Department of Integrative Biology, The University of Texas at Austin, Texas, USA; 2 Center for Computational Biology and Bioinformatics, The University of Texas at Austin, Texas, USA; 3 Department of Integrative Biology, Michigan State University, East Lansing, Michigan, USA; 4 Institute for Cellular and Molecular Biology, Institute for Neuroscience, The University of Texas at Austin, Texas, USA

**Keywords:** developmental hourglass, phylotypic stage, diversification, evo-devo

## Abstract

Despite life’s diversity, studies of variation often remind us of our shared evolutionary past. Abundant genome sequencing and analyses of gene regulatory networks illustrate that genes and entire pathways are conserved, reused, and elaborated in the evolution of diversity. Predating these discoveries, 19th-century embryologists observed that though morphology at birth varies tremendously, certain stages of vertebrate embryogenesis appear remarkably similar across vertebrates. In the mid to late 20th century, anatomical variability of early and late-stage embryos and conservation of mid-stages embryos (the “phylotypic” stage) was named the hourglass model of diversification. This model has found mixed support in recent analyses comparing gene expression across species possibly owing to differences in species, embryonic stages, and gene sets compared. We compare 186 microarray and RNA-seq data sets covering embryogenesis in six vertebrate species. We use an unbiased clustering approach to group stages of embryogenesis by transcriptomic similarity and ask whether gene expression similarity of clustered embryonic stages deviates from a null expectation. We characterize expression conservation patterns of each gene at each evolutionary node after correcting for phylogenetic nonindependence. We find significant enrichment of genes exhibiting early conservation, hourglass, late conservation patterns in both microarray and RNA-seq data sets. Enrichment of genes showing patterned conservation through embryogenesis indicates diversification of embryogenesis may be temporally constrained. However, the circumstances under which each pattern emerges remain unknown and require both broad evolutionary sampling and systematic examination of embryogenesis across species.

## Introduction

Significance

Vertebrates, like arthropods or flowering plants, establish a conserved body plan during development from which species-specific elaborations develop. Yet, the generalizable rules that direct this conservation of embryogenesis across distantly related species have remained controversial for 200 years. We present a quantitative analysis of the primary literature to determine support for hypothesized patterns of conservation of embryogenesis. We then perform a comparative analysis of 186 publicly available microarray and RNA-seq expression data sets covering embryogenesis in six vertebrate species spanning ∼420 Myr of evolution. We find strong support for temporal patterning in diversification of gene expression and conclude that broad evolutionary sampling and systematic examination of embryogenesis will enable increasingly powerful inferences about the rules directing conservation of vertebrate embryogenesis.

**Fig. 1:**
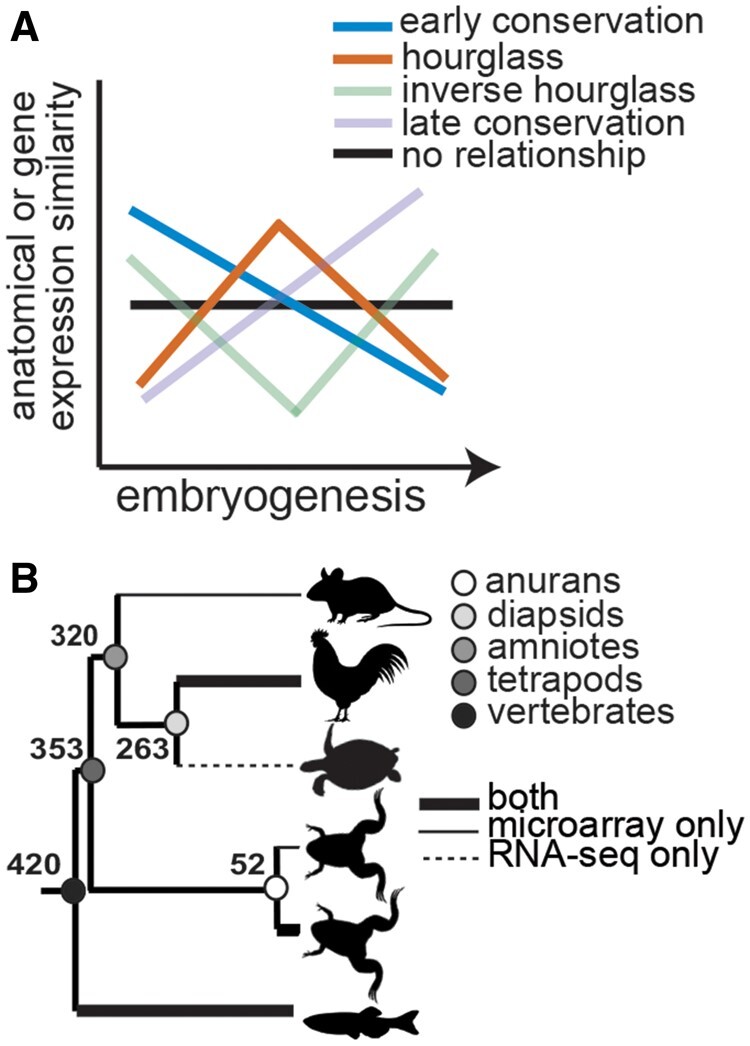
Anatomical and gene expression similarity predicted under different models of conservation through embryogenesis (*A*). Expression conservation was assessed using 186 publicly available microarray and RNA-seq data sets through embryogenesis across a phylogeny of six vertebrate species (*B*). Divergence times at each node are shown in millions of years.

During embryogenesis, a single-cell zygote develops into a multicellular, functional embryo. Given the complexity of this process and the astonishing diversity of resultant phenotypes, the similarities in the developmental processes and anatomy of embryogenesis across species are striking and have captivated the imagination of biologists for nearly two centuries (von Baer 1828). For example, vertebrates establish a highly conserved body plan (“*bauplan*”) from which species-specific variation and elaborations develop. Yet, whether there are generalizable rules that direct diversification of embryogenesis across distantly related species remains controversial (Richardson et al. 1997; Bininda-Emonds et al. 2003). Inspired by von Baer’s (1828) pioneering observations (reviewed in Sander and Schmidt-Ott 2004; Brauckmann 2008; Abzhanov 2013), one hypothesis suggests that early and late phases of embryogenesis are variable across species (owing to diversity and species specificity of reproductive modes and post-body plan elaboration, respectively), whereas anatomy of mid-embryogenesis is conserved (fig. 1). According to this “developmental hourglass” hypothesis (Elinson 1987), similarity of the mid-embryogenesis “phylotypic stage” (at the pharyngula stage: Ballard 1976, 1981; Sander 1983; Richardson 1995) reflects developmental constraints of body plan formation, including global signaling interdependence and interactions (Raff 1996; Galis and Metz 2001) and temporal and spatial patterns of *Hox* expression (Duboule 1994). Still others have hypothesized that development of later stages is dependent on early stages of embryogenesis and that this “developmental burden” results in highest conservation early in embryogenesis (Riedl 1978; discussed in Irie and Kuratani 2014) (fig. 1).

**Fig. 2:**
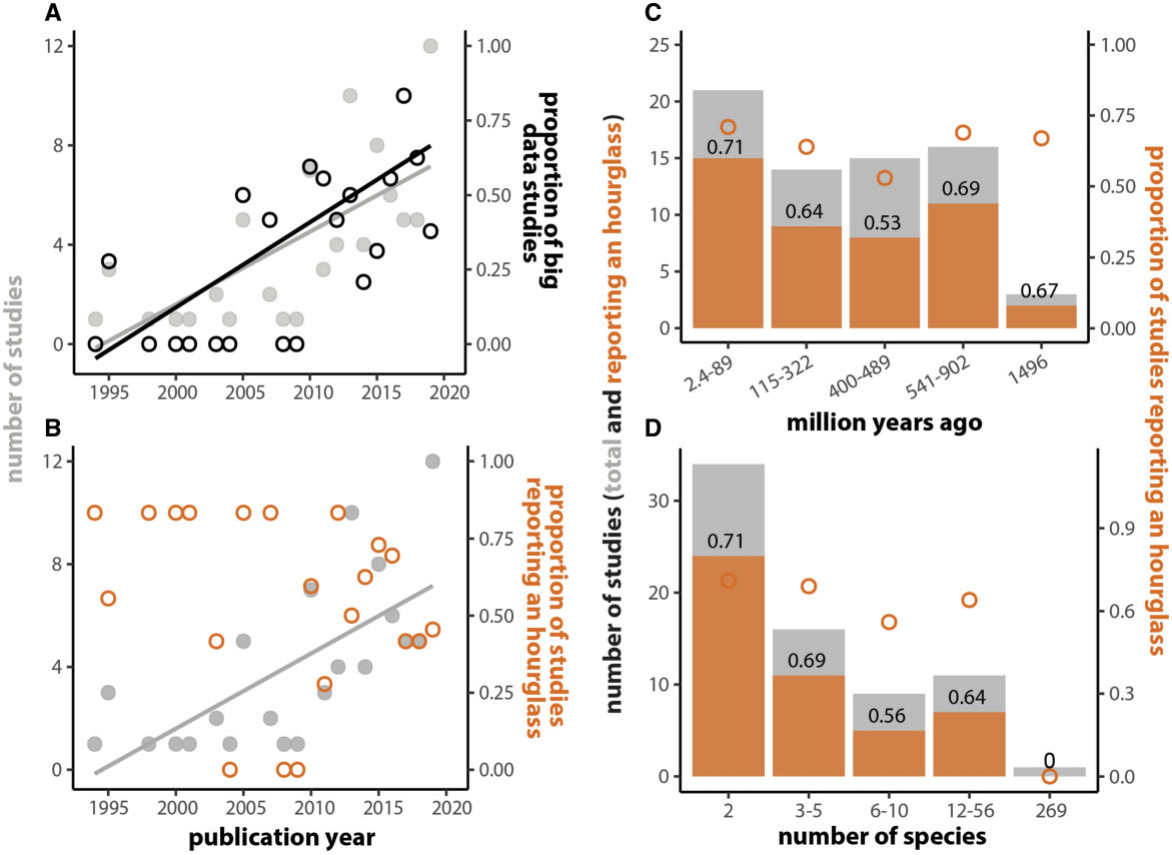
The number of studies testing the developmental hourglass and alternative hypotheses has increased considerably since the start of the new millennium (*A*, gray line: *r*^2^ = 0.5, *F*_(1, 19)_ = 21, *P* = 0.0002), driven in large part by an increase in the number of gene expression studies (*A*, black line: *r*^2^ = 0.27, *F*_(1, 19)_ = 8.3, *P* = 0.01). Despite this increased research effort, whether variation in embryogenesis follows an hourglass pattern has remained unresolved (*B*, orange points: *r*^2^ = 0.008, *F*_(1, 19)_ = 1.2, *P* = 0.29). Neither divergence time of the species compared (*C*), nor number of species included in any given study (*D*) affect whether an hourglass pattern is observed. Quantitative literature analysis of studies examining early embryonic development across species was carried out according to the PRISMA flow diagram (supplementary fig. 1, Supplementary Material online; Moher et al. 2009). Detailed methods are provided in supplementary material (supplementary tables S1 and S2 and fig. S1, Supplementary Material online).

Over the past decade, an increasing number of studies have leveraged the ever-growing genome-scale data and approaches to test omics-level predictions of the hourglass hypothesis and its underlying mechanistic basis (fig. 2*A* and supplementary fig. S1 and table S2, Supplementary Material online). Nevertheless, support for the hourglass model of development varies across studies and the question remains very much unsettled (fig 2*B*). A number of studies comparing gene expression variation through embryogenesis across species have found support for an increase in expression conservation mid-embryogenesis (Irie and Kuratani 2011, 2014; Yanai et al. 2011; Levin et al. 2012; Wang et al. 2013; Gerstein et al. 2014; Zalts and Yanai 2017). Surprisingly, one study comparing expression divergence of animals from different phyla reported an inverse hourglass—where expression differences were highest mid-embryogenesis (Levin et al. 2016)—although this analysis did not account for phylogenetic nonindependence (Dunn et al. 2018). Still others found that diversification in gene expression is not consistent with the hourglass model (Tian et al. 2013; Wu et al. 2019). von Baer’s (1828) original morphological observations were based on vertebrate embryos separated by ∼420 Myr of evolution; however, recent analyses varied in the evolutionary distances among species investigated (fig. 2*C*), sometimes even spanning more than one phylum (de Mendoza et al. 2013; Levin et al. 2016; Hu et al. 2017) and often testing predictions of the hourglass hypothesis in only one or two species (e.g., zebrafish, Domazet-Loso and Tautz 2010; soft-shell turtle and chicken, Wang et al. 2013; *Caenorhabditis elegans*, Zalts and Yanai 2017; fig. 2*D*). Taken together, even though numerous studies have used sophisticated –omics level analyses to examine embryogenesis across diverse species, we still lack conclusive molecular evidence of a developmental hourglass.

Any test of the developmental hourglass hypothesis faces several fundamental challenges. First, proper alignment of stages of embryogenesis across species is difficult. Despite several valiant attempts to overcome this difficulty (Bininda-Emonds et al. 2003; Mungall et al. 2012; Gerstein et al. 2014; Li et al. 2014), nomenclature and sampling conventions that often vary substantially across different model systems as well as widespread heterochrony of developmental events have prevented a satisfactory solution. Irie and Kuratani (2011) circumvented this challenge by directly comparing only a subset of well-defined stages across species, but it is unclear to what extent any observed temporal pattern might depend on the stages selected for comparison. Second, what constitutes an appropriate gene set to compare across species is very much unclear. Most studies to date have examined conservation of all expressed genes for which orthologs can be identified for all the taxa in the analysis. However, others have suggested that abundant expression of housekeeping genes may bias discovery of gene expression conservation across species (Piasecka et al. 2013). Third, it is clear that the genomic and developmental processes underlying even anatomically similar and homologous phenotypes can diverge via developmental drift or selective processes (de Beer 1971; Wagner 1989; True and Haag 2001; Wilkins 2002; McGary et al. 2010; Young and Wagner 2011). As a result, some authors have argued that studies examining the evolution of organismal phenotypes should focus on a core set of regulatory genes critical to the initiation of the specific developmental program of that character (designated as “kernels” by Davidson and Erwin 2006, or “Character Identity Networks, ChINs” by Wagner 2007). However, how to identify the relevant gene set (i.e., kernel or ChIN) that is fundamental for shared developmental processes of vertebrate embryogenesis is unclear. Finally, it is often not clear what the appropriate null expectation should be in comparative studies, as it may depend on the type of data available and the level of analysis (Dunn et al. 2018; Young and Hofmann 2019; Church and Extavour 2020).

Although numerous studies have reported patterned expression divergence (i.e., early conservation or hourglass patterns) through embryogenesis across species, what pattern is followed and whether that diversification pattern varies over evolutionary time remains unclear. Here, we perform a comparative analysis using 186 publicly available microarray and RNA-seq expression data sets covering embryogenesis in six vertebrate species spanning ∼420 Myr of evolution. We use an unbiased clustering approach to group stages of embryogenesis by transcriptomic similarity and ask whether gene expression similarity of clustered embryonic stages deviates from the null hypothesis that gene expression levels are invariant of developmental time. Second, we characterized the expression conservation pattern (i.e., early conservation, hourglass, inverse hourglass, late conservation, or no relationship) exhibited by each gene at each evolutionary node after correcting for phylogenetic nonindependence and ask whether the number of genes that fall into a given pattern deviates from a biologically meaningful null expectation. Finally, we discuss challenges of comparative analyses that rely on publicly available transcriptome data and suggest several novel approaches for future tests of the hourglass hypothesis.

## Results

### Variation in Gene Sets and Alignment of Embryonic Stage Clusters across Species and Gene Expression Profiling Technologies

We identified 1,626 and 1,782 one-to-one orthologs for the microarray and RNA-seq data in our analysis, respectively, consistent with other comparative gene expression studies spanning vertebrates (supplementary fig. S3, Supplementary Material online; 3,044 avian to human orthologs: Pfenning et al. 2014; 1,979 orthologs across Peromyscus mice, Microtus voles, Passeroid birds, Dendrobatid frogs, and Ectodini cichlids: Young et al. 2019). Interestingly, one-to-one orthologs from the microarray and RNA-seq data set were largely nonoverlapping with a total of 255 overlapping genes (15.7% and 14.3% of each set of orthologs, respectively). Similarly, one-to-one orthologs from microarray and RNA-seq data sets from the same species were largely nonoverlapping as well (∼15–20%, supplementary table S10, Supplementary Material online). Consistency of this overlap across species suggests that microarray and RNA-seq approaches may target distinct features of the transcriptome. Alternatively, nonoverlapping one-to-one orthologs may be a consequence of the sequence-based ortholog calling approach. Sequence-based ortholog calling clusters proteins by sequence similarity. Specifically, proteins whose sequences are more similar within than across species will be included as paralogs. Thus, different gene sets and different species (e.g., in the microarray and RNA-seq data sets) could result in different grouping of genes.

**Fig. 3.**
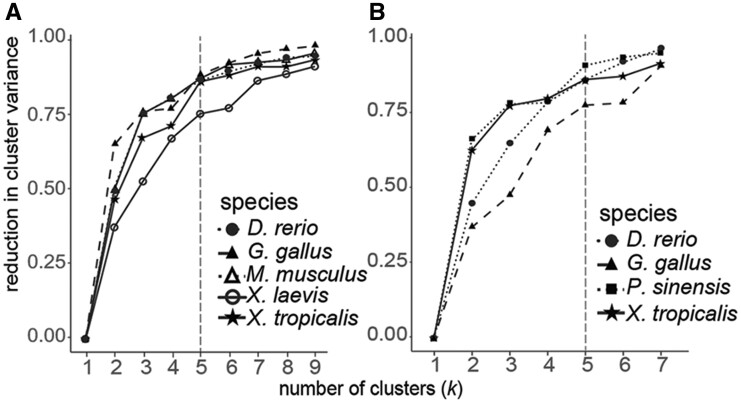
k-means clustering of the microarray (*A*) and RNA-seq (*B*) data sets analyzed. Reduction of within cluster variance increases as the number of cluster (*k*) increases. Gains asymptote at approximately *k *=* *5 (dashed line).

**Fig. 4:**
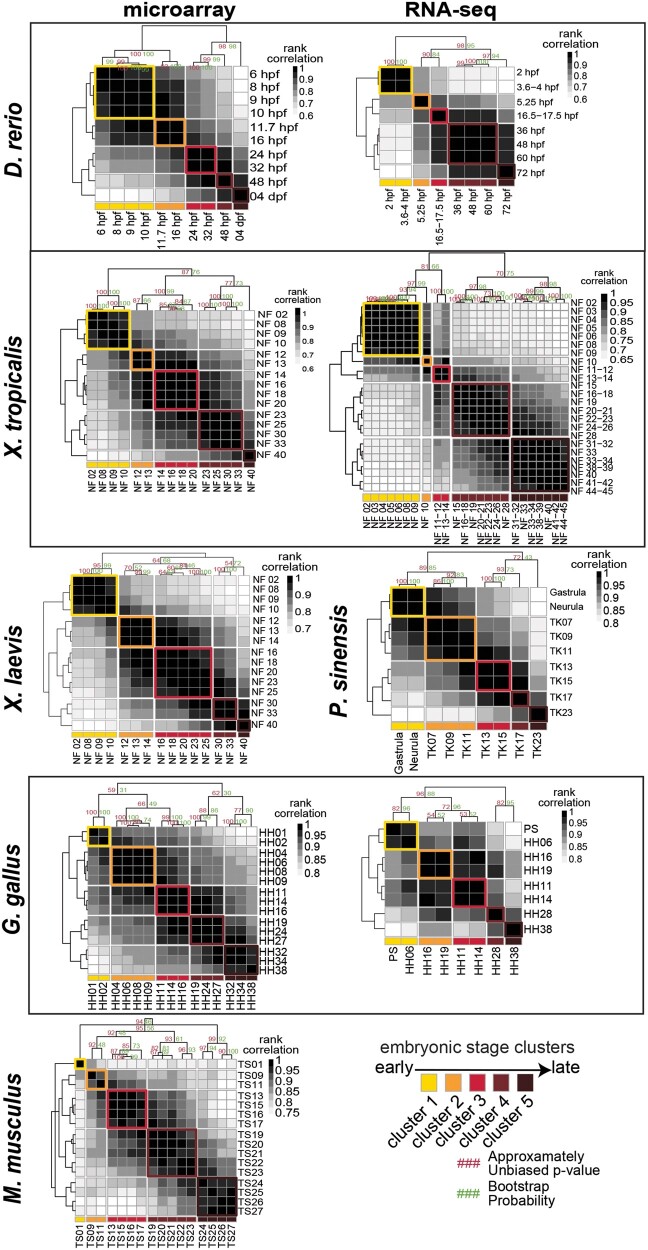
Spearman rank correlations were used to group stages into five clusters. Shown are all pairwise correlations of stages for all species and both gene expression profiling technologies. Grouping of stages is in indicated color.

**Fig. 5:**
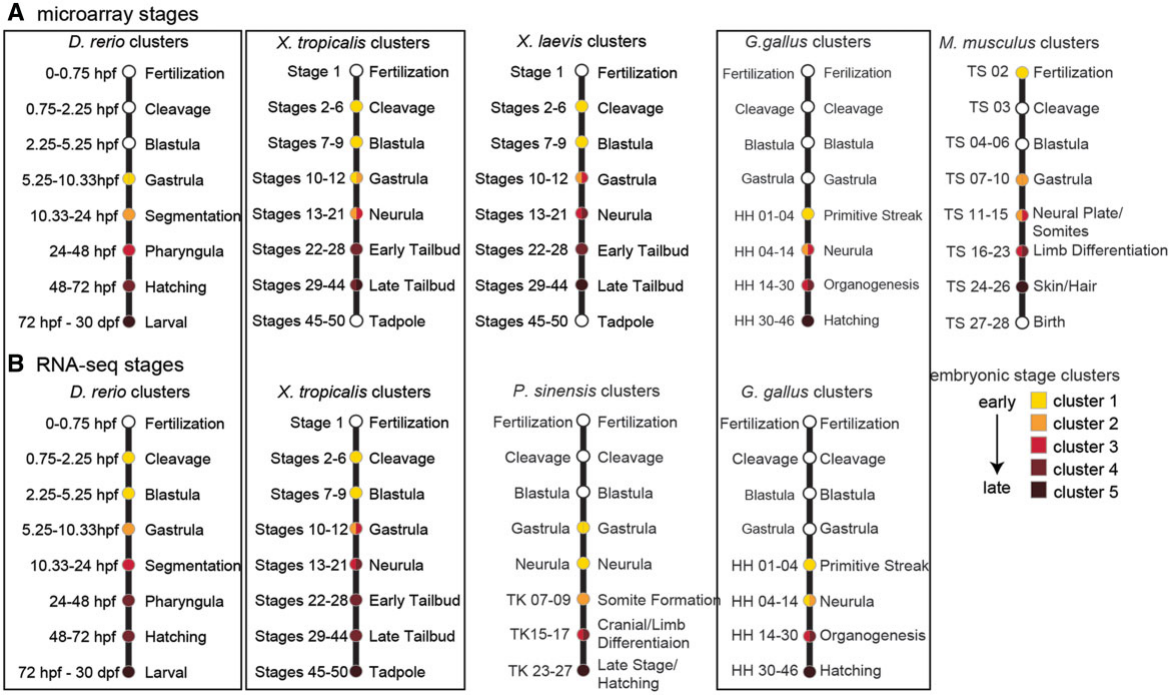
Overlap of embryonic stage cluster and major developmental events. Variation in sampling and heterochrony among species result in differences of developmental events captured by the available data across species and platforms. For three species, gene expression data sets were obtained from both microarray (*A*) and RNA-seq (*B*) platforms.

When we clustered the embryonic stages according to their gene expression patterns, we found that, for each species and gene expression profiling technology, a *k *=* *5 emerged as the number of clusters at which the reduction in within-clustervariance begins to asymptote (fig. 3). Importantly, the temporal order of stages across embryogenesis was maintained at *k *=* *5 clusters; however, inconsistent sampling across species as well as heterochrony across species resulted in some variation in the major events contained in each cluster of embryonic stages across species and between microarray and RNA-seq platforms (fig. 4). Stages contained in each cluster and a biological description of embryonic events are provided in figure 5 and supplementary tables S5 and S6, Supplementary Material online (microarray and RNA-seq, respectively). The number of stages collapsed into each cluster also varied across species and gene expression profiling technology. Because there was no apparent bias between cluster timing in embryogenesis and the number of stages included (fig. 5), we concluded that *k *=* *5 clusters was appropriate for downstream analyses.

### Conservation of Gene Expression at the Transcriptome Level

**Fig. 6:**
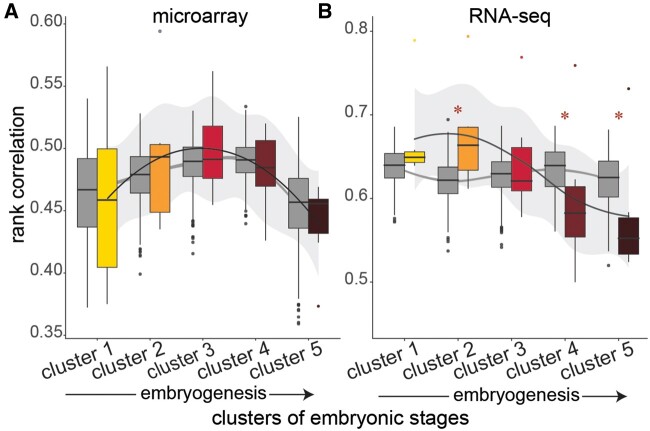
Spearman rank correlations for pairwise comparisons of species at each cluster of embryogenesis for microarray (*A*) and RNA-seq data (*B*). Gene expression correlations (as a measure of conservation) vary through embryogenesis for both microarray and RNA-seq data and show support for a developmental hourglass. Colored boxes indicate observed correlations; gray boxes indicate rank correlations after permutation analysis randomizing stage association with cluster. Asterisks indicate that the observed median correlation differs significantly from the null expectation at *P* < 0.01. Note that only the RNA-seq data showed a temporal pattern (consistent with the early conservation hypothesis) that differed from the null expectation (i.e., no temporal pattern present).

We then quantified interspecific gene expression correlation for each of the *k *=* *5 clusters of embryogenesis identified in figure 4. Clusters that are correlated across species are more conserved. We found that the pattern of pairwise rank correlations for all one-to-one orthologs (1,626 and 1,781 for microarray and RNA-seq, respectively) through embryogenesis differed for microarray and RNA-seq comparisons (fig. 6). In the microarray comparison, median pairwise rank correlations increased mid-embryogenesis, with clusters 2, 3, and 4 having the highest median correlation, and decreased early and late in embryogenesis, with cluster 5 having the lowest median, suggesting a developmental hourglass (fig. 6*A*). However, when compared with the null expectation of no gene expression conservation above chance, generated by permuting the cluster assignment of each stage, the observed pattern did not differ from the null (cluster 1: observed median Spearman’s ρ_o_ = 0.46, permutation Spearman’s ρ_p_ = [0.37–0.54], *P* = 0.43; cluster 2: ρ_o_ = 0.49, ρ_p_ = [0.4–0.53], *P* = 0.26; cluster 3: ρ_o_ = 0.49, ρ_p_ = [0.42–0.53], *P* = 0.47; cluster 4: ρ_o_ = 0.48, ρ_p_ = [0.44–0.53], *P* = 0.35; cluster 5: ρ_o_ = 0.46, ρ_p_ = [0.36–0.52], *P* = 0.49). In the RNA-seq comparison, median pairwise rank correlations increased to its highest median score at cluster 2 and dropped through later stages of embryogenesis 3, with cluster 5 having the lowest median (fig. 6*B*). When compared with the null expectation, median pairwise rank correlation of cluster 2 was significantly higher and correlations of clusters 4 and 5 were significantly lower than expected by chance, providing strong support for conservation of mid-embryogenesis and divergence late in embryogenesis (cluster 1: ρ_o_ = 0.65, ρ_p_ = [0.56–0.68], *P* = 0.24; cluster 2: ρ_o_ = 0.67, ρ_p_ = [0.52–0.69], *P* = 0.005; cluster 3: ρ_o_ = 0.62, ρ_p_ = [0.51–0.68], *P* = 0.49; cluster 4: ρ_o_ = 0.57, ρ_p_ = [0.57–0.68], *P*  = 0.003; cluster 5: ρ_o_ = 0.53, ρ_p_ = [0.59–0.69], *P* = 0.008). Although this pattern appears consistent with the developmental hourglass, conserved cluster 2 contains gastrula stages (*D. rerio* and *X. tropicalis*) and neurula stages (*G. gallus*) which occur earlier in embryogenesis than what is typically considered the phylotypic stage (i.e., the pharyngula stages).

### Enrichment and Overlap of Expression Conservation Patterns across the Phylogeny

**Fig. 7:**
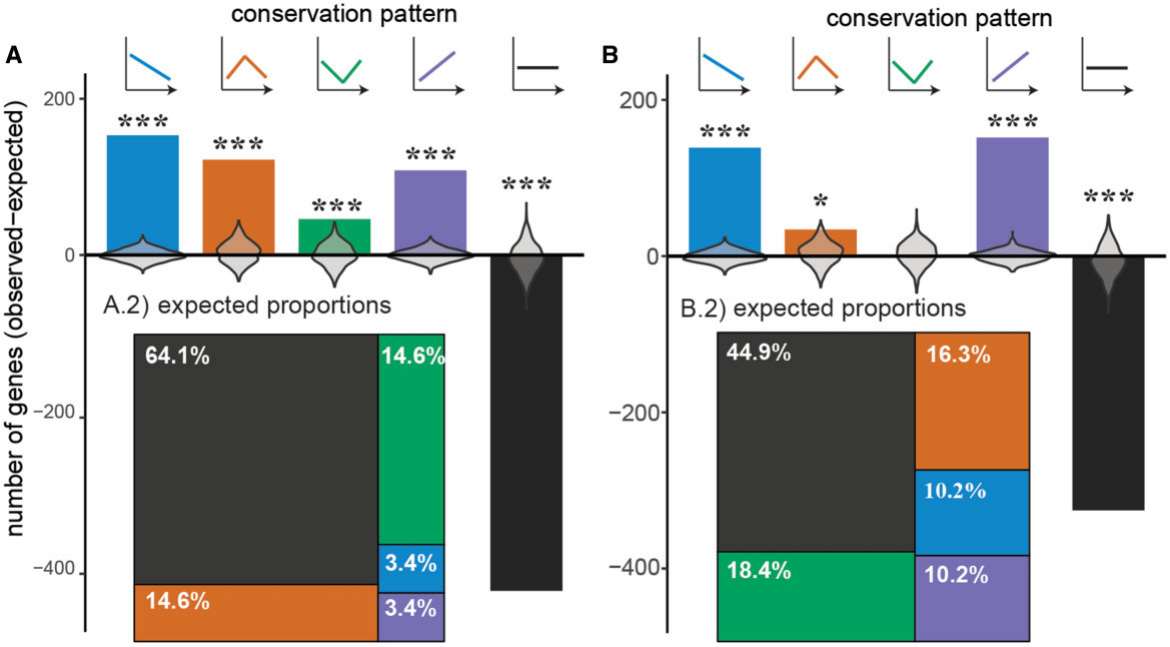
Enrichment of temporally patterned gene expression conservation across vertebrates. The expected number of genes for a given temporal expression pattern is determined by the proportion of trajectories of each conservation pattern (A.2: microarray; B.2: RNA-seq). The proportion of trajectories differed across conservation patterns and between platforms (A.2 and B.2). The enrichment/depletion values from permutation analysis (1,000 iterations) are shown as violin plots (A.1 and B.1). The number of genes associated with all conservation patterns were enriched compared with random expectation, except for the inverse hourglass (RNA-seq only), whereas the number of genes with no relationship between expression conservation across embryogenesis (A.1: microarray; B.1: RNA-seq) was significantly less than expected by chance. For all plots, colors indicate the expression conservation pattern. Enrichment at other evolutionary nodes (tetrapods, amniotes, and anurans) are provided in supplementary figure S4, Supplementary Material online.

Next, we used ctsGE time series analysis (Sharabi-Schwager and Ophir 2019) to characterize patterns of gene expression conservation across embryogenesis at each phylogenetic node. For the microarray data set, gene conservation scores yielded a total of 90, 90, 87, 89 patterns of expression conservation in anurans, amniotes, tetrapods, and vertebrates, respectively. For the RNA-seq data set, gene conservation scores yielded a total of 90, 51, and 49 patterns in amniotes, tetrapods, and vertebrates. We calculated the expected number of genes for each pattern as equivalent to the proportion of total patterns (fig. 7, treemap insets; Tennekes 2017). Across vertebrates, with the exception of inverse hourglass in the RNA-seq analysis and time invariance in both platforms, all patterns were exhibited by significantly more genes than expected by chance (fig. 7). This enrichment of genes across all conservation patterns and the depletion of time-invariant genes deviated significantly from the null expectation (microarray: early conservation *P* < 10e-4, hourglass *P* < 10e-4, inverse hourglass *P* < 10e-4, late conservation *P* < 10e-4, time invariance *P* < 10e-4; RNA-seq: early conservation *P* < 10e-4, hourglass *P* = 0.02, inverse hourglass *P* = 0.4, late conservation *P* < 10e-4, time invariance *P* < 10e-4). Enrichment/depletion of genes for each conservation pattern across anurans (microarray only), amniotes, and tetrapods are provided in supplementary figure S4, Supplementary Material online. *P* < 10e-4 indicates that none of the 1,000 permutation iterations resulted in enrichment/depletion at or above/below the observed level.

Finally, we compared conservation pattern assignment for the 255 one-to-one orthologous gene groups shared between microarray and RNA-seq gene sets and found that across platforms gene expression patterns are not concordant at the major phylogenetic nodes. Specifically, only 25% of genes (63/255) in amniotes, 19% (49/255) in tetrapods, and 20% (52/255) in vertebrates exhibited concordant expression conservation patterns.

## Discussion

In the present study, we examined patterns of expression diversification throughout embryogenesis in six vertebrate species using a comparative analysis of 112 microarray and 74 RNA-seq data sets (fig. 1*B*). First, using an unbiased approach, we clustered stages of embryogenesis within species and compared expression conservation of those clusters across species (figs. 3–5; supplementary tables S5 and S6, Supplementary Material online). This approach allowed for the inclusion of all available stages of embryogenesis and removed bias that could result from comparing only selected stages. Second, we used a permutation analysis to generate a null expectation. We observed conservation estimates against this null expectation to characterize transcriptome-level diversification through embryogenesis across species (fig. 4). Finally, we characterized the expression conservation of each gene at each node of the phylogeny (fig. 1*B* and supplementary fig. S2, Supplementary Material online) to examine how expression conservation patterns vary through evolutionary time (fig. 6).

Over the past decade, the debate of whether diversification of embryogenesis follows generalizable rules has been reinvigorated by the ability to test predictions of the hourglass, developmental burden, and other hypotheses (fig. 1*A*) on a genomic scale. Enabled by the increase in “omics-level” data and next-generation sequencing accessibility, a number of studies have by now explored patterns of diversification in gene expression though embryogenesis across species (fig. 2). These studies have found mixed support for the hourglass and other models of diversification across species (fig. 2*B*–*D*; reviewed in Irie 2017; Liu and Robinson-Rechavi 2018). Differences among studies could reflect differences in species compared as some studies span phyla (de Mendoza et al. 2013; Levin et al. 2016; Hu et al. 2017) and others are restricted to internal nodes of the vertebrate phylogeny (e.g., amniotes; Wang et al. 2013). However, our quantitative literature analysis did not indicate an effect of divergence time on the characterization of an hourglass model of divergence (fig. 2*C*). Alternatively, selection of developmental stages or specific gene sets to compare may lead to different results and interpretations. Finally, studies that compare gene expression similarity at the transcriptome level rarely test against a null hypothesis (Young and Hofmann 2019). Such a test is critical because the degree of variation expected through developmental stages across species is unknown (Church and Extavour 2020).

Aligning stages of early animal development is complicated by taxon-specific sampling practices as well as pervasive heterochrony in developmental events across distantly related species, leading some researchers to question the validity of anatomical hourglass hypotheses (Bininda-Emonds et al. 2003). We found that although expression varies in similar ways through embryogenesis across species, stages of embryogenesis did not always consistently cluster together within species (figs. 3 and 4 and supplementary tables S5 and S6, Supplementary Material online). These differences likely reflect both biological variation in the molecular timing of developmental events, technical variation in sampling procedures across species, and a lack of available data sets particularly at early embryonic stages. Because clustering has the advantage of being unbiased, and no systematic bias in sampling was apparent, we moved forward by comparing gene expression at each embryonic stage cluster across species. Future studies with systematic sampling of embryogenesis across species could disentangle the source (biological or technical) of variation in stage clustering across species.

**Fig. 8:**
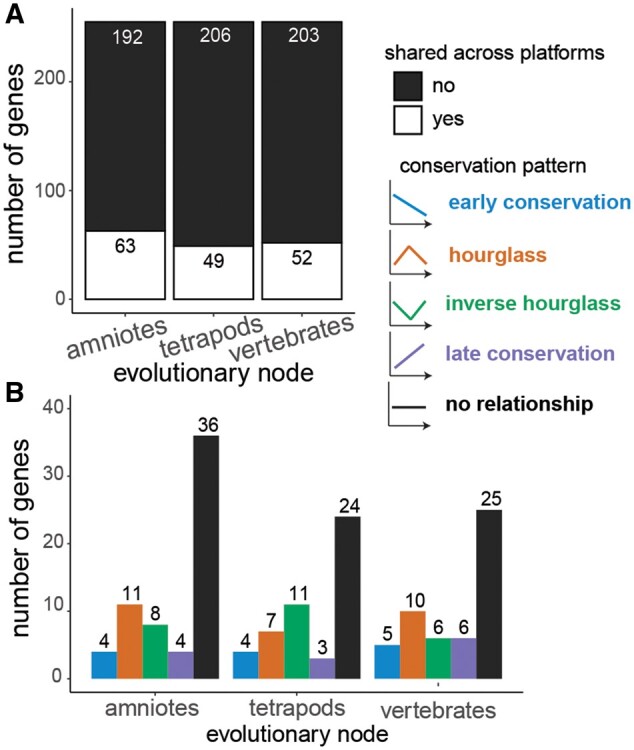
A total of 255 one-to-one orthologs shared between microarray and RNA-seq data sets largely differed in expression conservation pattern (*A*). Most genes with shared expression conservation patterns were “no relationship” genes followed by hourglass or inverse hourglass genes (*B*).

Consistent with the mixed support for the hourglass and other models of developmental divergence (e.g., early conservation or inverse hourglass) found across studies, our comparisons of expression variation in all one-to-one orthologs present across species in the microarray and RNA-seq data yielded significant but different patterns. Specifically, the observed gene expression correlations (as a measure of conservation) differed through embryogenesis for both microarray and RNA-seq, yet only the RNA-seq data showed a pattern that differed significantly from the null expectation that gene expression levels should be invariant of developmental time (fig. 6). For the RNA-seq data sets, we found a significant increase in expression correlation over the null expectation in developmental time cluster 2 followed by a significant reduction in expression correlation in the later clusters 4 and 5. Though clusters 1 and 2 display similar gene expression correlations, suggesting an early conservation pattern, cluster 1 does not significantly differ from the null expectation. Inconsistencies between the microarray and RNA-seq data sets could result from differences in taxon sampling (fig. 1*B*), the embryonic stages and resulting clusters that were included (figs. 4 and 5), and/or systematic differences in which aspects of the transcriptome were captured by these distinct platforms. In fact, transcriptome-level gene expression profiles quantified using RNA-seq and microarray technologies have been shown to be correlated especially for highly expressed genes, but variation between technologies is also commonly reported (Marioni et al. 2008; Malone and Oliver 2011; Trost et al. 2015). Whether these differences reflect superiority of one technology over the other is unclear. Instead technical differences between the two approaches may capture different elements of the transcriptome, in which case the two approaches should be viewed as complementary (Kogenaru et al. 2012). In our data sets, we found little overlap of one-to-one orthologs between microarray and RNA-seq, with only ∼15–20% of the genes contained in both analyses. Further, of those one-to-one orthologs contained in both microarray and RNA-seq data sets, only ∼20% shared conservation pattern assignments (fig. 8). This illustrates a major challenge for comparative analyses, like our present study, that aim to capitalize on the vast amounts of publicly available transcriptome data sets to test biological hypotheses.

Inconsistency across studies and gene expression profiling technology also suggests that comparing the whole transcriptome may not be appropriate. Specifically, patterns of expression conservation at the whole transcriptome level may be biased by abundant and constitutively expressed genes (e.g., see Piasecka et al. 2013). Whole genome approaches have the potential of being unbiased, which allows for the identification of novel gene associations with phenotypes and/or gene interactions that would be missed using a candidate gene approach. However, we know that variation is not equivalent across levels of biological organization. For example, studies comparing mRNA and protein levels have found that overall protein abundances are moderately correlated mRNA abundances (Foss et al. 2007; Fu et al. 2009; Ghazalpour et al. 2011; Vogel and Marcotte 2012; Liu et al. 2016; Fortelny et al. 2017; Buccitelli and Selbach 2020). Although some technical variation between protein and mRNA quantification approaches impact this correlation, biological differences (e.g. temporal dependencies and spatial variation in transcription and translation) illustrate the complementarity of inferences made at different biological levels (Buccitelli and Selbach 2020). Further, studies of character homology have shown that even anatomically and physiologically similar homologies can differ in underlying developmental and molecular mechanisms (Wagner 1989; Wilkins 2002; McGary et al. 2010; Young and Wagner 2011). To address this issue, we also used a time series analysis to characterize the conservation pattern of each gene at each node of the phylogeny (fig. 7), in addition to a transcriptome-level comparisons through embryogenesis across species. Across vertebrates, we observed a significant enrichment of all patterned conservation models (early conservation, hourglass, inverse hourglass, and late conservation) above the expected number and a large depletion of genes whose expression was invariant of developmental time (fig. 7 and supplementary fig. S4, Supplementary Material online) in both microarray and RNA-seq data sets. Both the enrichment of genes exhibiting different patterns and the proportion of overall genes with distinct patterns varied across evolutionary nodes (e.g., tetrapods, amniotes, and anurans, fig. 1*B*) and between gene expression profiling technologies (supplementary fig. S4, Supplementary Material online). These results suggest that divergence in gene expression through embryogenesis may depend on the evolutionary distance covered by any given analysis. Follow-up studies, including phylogenetic comparative analyses of both closely and distantly related species are needed to better understand these patterns and their implications for generating biological diversity. In addition, time series analyses that characterize expression divergence of individual genes or gene sets should be used to test hypothetical mechanisms of constraint. For example, we might ask whether regulatory interactions or temporal and spatial expression patterns of a gene follow a correlated dynamic pattern through embryogenesis. Finally, here, we focus on expression conservation in one-to-one orthologs; however, patterns of genome evolution that can result in complexities in gene orthology (e.g., gene duplication and loss) should be further explored to fully characterize general patterns in evolution of complex phenotypes such as embryogenesis.

## Conclusions

Taken together, our results provided strong support for a patterned embryonic gene expression diversification across vertebrate species. However, the gene groups and evolutionary nodes under which each pattern emerges remain unknown. By combining unbiased clustering of embryonic stages and explicit tests against a null hypothesis our research demonstrates a critical need for broad evolutionary sampling and systematic examination of developmental stages across species to better characterize gene expression diversification in embryogenesis.

## Materials and Methods

### Obtaining and Preprocessing Genome-Wide Gene Expression Data from Public Repositories

Gene expression profiles through embryogenesis were obtained from publicly available repositories for six vertebrate species. In total, 112 microarray data sets from five species and 74 RNA-seq data sets from four species were included in the analysis. Gene expression profiling methods for each study are provided in supplementary tables S3 and S4, Supplementary Material online (microarray and RNA-seq, respectively). Developmental time points included for each species and gene expression profiling technology are detailed in supplementary tables S5 and S6, Supplementary Material online (microarray and RNA-seq, respectively). Data sets include: zebrafish, *Danio rerio*, a microarray data set (ten embryonic stages: Saric et al. 2005) and an RNA-seq data set (seven embryonic stages: Yang et al. 2013); chicken, *Gallus*, a microarray data set (15 embryonic stages: Irie and Kuratani 2011) and an RNA-seq data set (8 embryonic stages: Wang et al. 2013); a Chinese soft-shell turtle, *Pelodiscus sinensis*, RNA-seq data set (9 embryonic stages: Wang et al. 2013); two mouse, *Mus musculus*, microarray data sets (eight embryonic stages: Irie and Kuratani 2011; 11 embryonic stages: Xue et al. 2013); an African clawed frog, *Xenopus laevis*, microarray data set (15 embryonic stages: Yanai et al. 2011); a Western clawed frogs, *Xenopus tropicalis*, microarray data set (15 embryonic stages: Yanai et al. 2011) and an RNA-seq data set (23 embryonic stages: Tan et al. 2013). *Daniorerio* expression data at the zygote developmental stage (0.25 hpf) was excluded because of likely abundance of maternal transcripts, and time points after 4 dpf were excluded due to substantial completion of the developmental program (after Kimmel et al. 1995; Yang et al. 2013).

### Preprocessing Microarray and RNA-Seq Data

Affymetrix and Agilent microarray data were imported directly using the R packages *simpleaffy* and *limma*, respectively (Wilson and Miller 2005; Ritchie et al. 2015). For both microarray platforms, preprocessing consisted of RMA background correction with quantile normalization (Irizarry et al. 2003). This information was automatically attained by *limma* for Agilent data sets. For Affymetrix and Agilent data, probe sets that mapped to multiple genes or no genes at all were excluded from further analysis. All expression values were then transformed to log-scale using the function *log_2_*(*x*) (“Log2-transformed”). For RNA-seq data, SOLiD data were converted to fastq using the Sequence Read Archive toolkit, and raw reads were checked for quality using FastQC (Andrews 2010). All data sets were of good quality with less than 10% adaptor contamination; thus, no trimming was required. After quality control, raw reads were pseudoaligned to species-specific reference transcriptomes using Kallisto to produce read counts (Bray et al. 2016). Read counts were transformed to transcripts per million mapped reads. The package *biomaRt* was used to assign corresponding ENSEMBL gene ID(s) to each Affymetrix probe set and RNA-seq transcript (Durinck et al. 2009). For microarray data, the signals of all probe sets mapping to the same gene were averaged. For RNA-seq counts mapped to different transcripts of the same gene were summed. Expression of each gene was averaged across biological replicates for developmental time point. These were the expression values used for downstream analysis.

### Ortholog Calling

To identify orthologous genes across taxa, we used the sequence-based ortholog calling software package OrthoMCL (Li et al. 2003) for microarray data and FastOrtho (Wattam et al. 2013; an implementation of OrthoMCL) for RNA-seq data. Predicted protein sequences of the reference genomes were organized into orthologous gene groups based on sequence similarity. For each reference proteome, protein and corresponding gene ids were grouped as paralogs when sequence similarity was higher among genes within species than between species. To facilitate downstream analysis of expression conservation across taxa, we removed any orthologous gene groups containing paralogs or losses/absences in one or more species. The resulting one-to-one orthologs were used for all downstream analyses (supplementary tables S7 and S8, Supplementary Material online, microarray and RNA-seq, respectively). To assess similarity in microarray and RNA-seq comparison, we compared the one-to-one ortholog sets of three species (zebrafish, chicken, and the Western clawed frog).

### Clustering of Embryonic Stages

We used transcriptomic similarity to classify the embryonic stages of each species in an unbiased manner. First, we determined the number of clusters using an elbow plot method. Specifically, for each species and gene expression profiling technology, we performed *k*-means clustering using gene expression for all developmental stages. We varied the number of clusters from 1 ≤ *k *≤* *9 for microarray and 1 ≤ *k *≤* *7 for RNA-seq and computed the sums of squares error (SSE, or variance within cluster) for all *k*. To determine an appropriate number of clusters, we used 1) the “elbow” effect (or determined the *k* at which additional cluster no longer results in a large reduction in SSE) and 2) determined the *k* where clustering of groups maintained temporal order of embryogenesis (i.e., no late stages cluster with early rather than other late stages). Other unsupervised soft clustering approaches (e.g., fuzzy c-means clustering; Hastie et al. 2000; Futschik and Carlisle 2005; Kumar and Futschik 2007) may be useful for future studies aimed at clustering time series gene expression data. Second, to generate clusters of embryonic stages for each species, we hierarchically clustered stages of embryogenesis by similarity in gene expression measured as Spearman’s rank correlations. The resulting dendrograms were partitioned into five groups to determine stage clusters. Description of developmental events were obtained from species-specific references including: zebrafish (Kimmel et al. 1995), chicken (Hamburger and Hamilton 1992), softshell turtle (Tokita and Kuratani 2001), both *Xenopus* species (Nieuwkoop and Faber 1994), and mouse (Graham et al. 2015). For clusters containing more than one embryonic stage, an expression mean was used as the representative expression for that cluster for the remaining analyses.

### Comparing Transcriptomes through Embryogenesis across Species

For both microarray and RNA-seq data, we assessed transcriptomic similarity at early, middle, and late phases of embryogenesis across species by calculating the Spearman’s rank correlation for all pairwise comparisons of species for each of the five clusters of embryonic stages. For microarray data, we excluded one frog (*X. laevis*) from the pairwise comparisons to prevent biasing the outcome as a consequence of high correlations in gene expression between the two congeneric anuran species at each cluster of embryonic stages. Due to the high correlations between these two species, similar results were recovered when *X. tropicalis* was removed from the analysis instead of *X. laevis*. We used permutation analysis to assess whether correlations are higher or lower than expected by chance. Specifically, for each species we randomly assigned stages to a cluster maintaining the original number of stages included in the observed cluster and computed the rank correlation for all pairwise species comparison. We conducted 1000 permutations and assessed significance by comparing the observed rank correlation to distribution of rank correlations generated by permutation analysis. Permutation *P* values were defined as the percentile of the observed median Spearman’s rho in the distribution of permuted Spearman’s rho values. Because correlation coefficients that are either greater or smaller than expected by chance (as determined by a two-tailed test) were of equal interest, we used the empirical cumulative distribution function in R to calculate both the percentile and one-percentile rank and report the lower of the two values.

### Characterizing Expression Conservation of Each Gene through Embryogenesis across Vertebrates

To assess conservation of gene expression for each gene at each cluster of embryonic stages and each node, we calculated a difference in expression rank scaled by the divergence time between the groups (supplementary fig. S2, Supplementary Material online). At each node of the phylogeny, we characterized patterns of expression conservation across embryogenesis using the R package Clustering of Time Series Gene Expression Data (*ctsGE*; Sharabi-Schwager and Ophir 2019). Using *ctsGE*, gene conservation scores of each gene were median scaled and converted into conservation indices. For each gene, at each cluster of embryonic stages, the standardized values indicate the median absolute distance of that gene from its median conservation score. These standardized values were then converted to index values that indicate whether gene expression conservation was above (1), below (−1), or within (0) the cutoff range (±0.7) of the median value at each time step (here cluster of embryonic stages). Optimal cutoff range was determined using the default setting that tests cutoffs between 0.5 and 0.7, in increments of 0.05, toward assigning an equal number of genes to each index. Each index of expression conservation across embryogenesis was assigned to a conservation pattern based on median transitions across the assigned significance cutoff. For early conservation: similarity decreases through embryogenesis; hourglass: similarity increases and then decreases; inverse hourglass: similarity decreases and then increases; late conservation: similarity increases; invariant: similarity does not vary or does not follow other conservation patterns through embryogenesis (fig. 1*A*). Indices assignments are provided in supplementary table S9, Supplementary Material online.

To examine enrichment of genes in each conservation pattern, we first calculated the proportion of indices in each conservation pattern and defined the expected number of genes as the equivalent proportion of total genes. We determined significance of enrichment/depletion of genes exhibiting each pattern using a permutation analysis. For each gene, we first randomized the order of conservation scores across the clusters of embryonic stages. Second, we characterized conservation trajectory using ctsGE and the index assignment rules described above. Finally, for each iteration we calculated enrichment/depletion of genes compared with the random expectation for each conservation pattern. We repeated this permutation 1000 times for each node and gene expression profiling technology and assessed by comparing the observed enrichment/depletion to the null distribution generated by the permutation analysis. Permutation *P* values were defined as the probability of obtaining enrichment/depletion of genes at or above/below the observed number in the permutation set as described above.

## Supplementary Material

Supplementary data are available at *Genome Biology and Evolution* online.

## Supplementary Material

evab160_Supplementary_Data
