# Supplementary material for: Comparative Transcriptomics Reveals Distinct Patterns of Gene Expression Conservation through Vertebrate Embryogenesis
Source: Genome Biol Evol. 2021 Jul 10;13(8):evab160. doi: 10.1093/gbe/evab160 (PMC8358226; doi:10.1093/gbe/evab160)
Supplement: evab160_Supplementary_Data [file evab160_supplementary_data.zip › Chanetal2021_GBE_Supp_revision2.pdf]

Supplementary Information:

Comparative transcriptomics reveals distinct patterns of gene expression conservation through vertebrate embryogenesis

Megan E. Chan[1,2], Pranav S. Bhamidipati[1,2], Heather J. Goldsby[3], Arend Hintze[3], Hans A. Hofmann[1,2,4], and Rebecca L. Young[1,2,*]

Supplementary Table 1. Studies examined and excluded from the literature analysis in Fig. 2.

Supplementary Table 2. Studies and data included in literature analyses Fig. 2.

Supplementary Table 3. Summary of microarray datasets including RNA preparation, microarray platform, and hybridization methods.

species	# of stages	microarray platform	RNA extraction	hybridization probes	doi or link
D. rerio	10	Affymetrix GeneChip	total RNA	Zebrafish Genome A-AFFY-38 Array	https://www.ebi.ac.uk/arrayexpress/experiments/E-TABM-33/
G. gallus	15	Affymetrix GeneChip	total RNA	Mouse Genome 430 2.0 Array; Chicken Genome Array; X. laevis Genome 2.0	10.1038/ncomms1248
M. musculus	11	Affymetrix GeneChip	total RNA	Mouse Genome 430 2.0	10.1186/1471-2164-14-568
	8	Affymetrix GeneChip	total RNA	Mouse Genome 430 2.0 Array; Chicken Genome Array; X. laevis Genome 2.0	10.1038/ncomms1248
X. laevis	15	Agilent Single-channel Gene Expression Microarray	total RNA	X. laevis custom Agilent 44k arrays (i.e., 4 blocks of 44k probes), 3 probes per orthologous gene	10.1016/j.devcel.2011.03.015
X. tropicalis	15	Agilent Single-channel Gene Expression Microarray	total RNA	X. tropicalis custom Agilent 44k arrays (i.e., 4 blocks of 44k probes), 3 probes per orthologous gene	10.1016/j.devcel.2011.03.015

Supplementary Table 4. Summary of RNA-seq data sets including RNA preparation, platform, and sequencing methods

species	# of stages	sequencing platform	RNA purification	paired or single-end	doi
D. rerio	7	SOLiD	Poly(A)	paired-end	10.1371/journal.pone.0064058
G. gallus	8	Illumina HiSeq	Poly(A)	single-end	10.1038/ng.2615
P. sinensis	9	Illumina HiSeq	Poly(A)	single-end	10.1038/ng.2615
X. tropicalis	23	Illumina HiSeq	Poly(A)	paired-end	10.1101/gr.141424.112

Supplementary Table 5. Description and clustering of embryonic stages in species with microarray data

Supplementary Table 6. Description and clustering of embryonic stages in species with RNA-seq data

Supplementary Table 7. Orthologous gene groups for microarray datasets

Supplementary Table 8. Orthologous gene groups for RNA-seq datasets

Supplementary Table 9. Gene conservations pattern indices

Supplementary Table 10. Number and percentage of overlapping one-to-one orthologs in microarray and RNA-seq datasets

**Species**	**# of overlapping orthologs**	**% of orthologs (microarray)**	**% of orthologs (RNA-seq)**
Gallus gallus	264	18.7%	18.0%
Xenopus tropicalis	345	21.2%	19.2%
Danio rerio	335	20.6%	16.2%

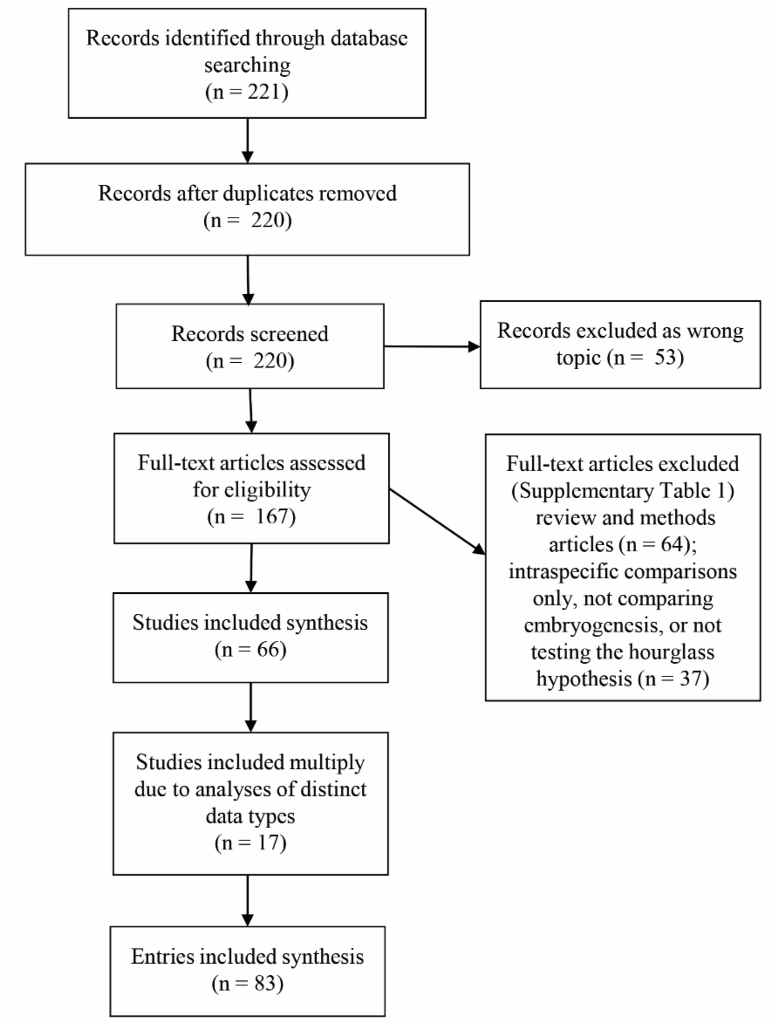

Supplementary Figure S1. Modified PRIMSA (Moher et al., 2009) flow chart. 220 unique articles retrieved from Web of Science using the following search criteria topic (TS) equals "phylotypic" OR "developmental AND hourglass" on March 25, 2020. We removed 53 articles that were not about the developmental hourglass hypothesis (e.g., in microbiology, "phylotypic" is sometimes used to classify a group of organisms by their phenetic relationship). We removed an additional 101 papers from the analysis including: 64 methods, modeling, opinion pieces, and review articles that did not include data analysis, 37 articles that included intraspecific comparisons only, focused on individual phenotypes (e.g., craniofacial morphology, brain, limbs, and heart) rather than embryogenesis, or do not test the hourglass hypothesis. A rationale for exclusion for each paper is provided in Supplementary Table 1. For each of the remaining 66 unique articles (Supplementary Table 2), the pattern of variation (early conservation, hourglass, inverse hourglass, late conservation, or no relationship) is reported for the type of data analyzed.

Data types were classified as molecular evolution, gene expression, morphological, regulatory, or genomic/proteomic. Gene expression studies include those comparing expression at the transcriptome-level (e.g., microarray and mRNA sequencing approaches) and candidate gene level. Molecular evolution studies characterize patterns of sequence evolution and associated evidence of selection at the whole genome or candidate gene level. Morphological studies compare anatomy and timing and order of development events. Regulatory studies focus on conservation of regulatory elements, miRNA, or chromatin modifications. Genomic/Proteomic studies characterize patterns of genome architecture (e.g., gene and gene family duplication) or protein interactions. Seventeen studies included analyses of data from two or more different levels of biological organization (e.g., molecular evolution and transcriptomics). These analyses were treated as independent, resulting in 83 entries total. Three studies compared species with unresolved divergence times and seven studies made comparisons between one species and more generalized groups (e.g., phylostratigraphy). These articles were excluded from divergence time comparisons in Fig. 2C. Finally, the same seven studies focused on only one species and, thus were excluded from the number of species comparison in Fig. 2D. Divergence time estimates were obtained from TimeTree (Kumar et al., 2017). A linear regression model was used to assess increases in research effort and gene expression-based studies and to test whether an increased proportion of studies find support for the hourglass hypothesis.

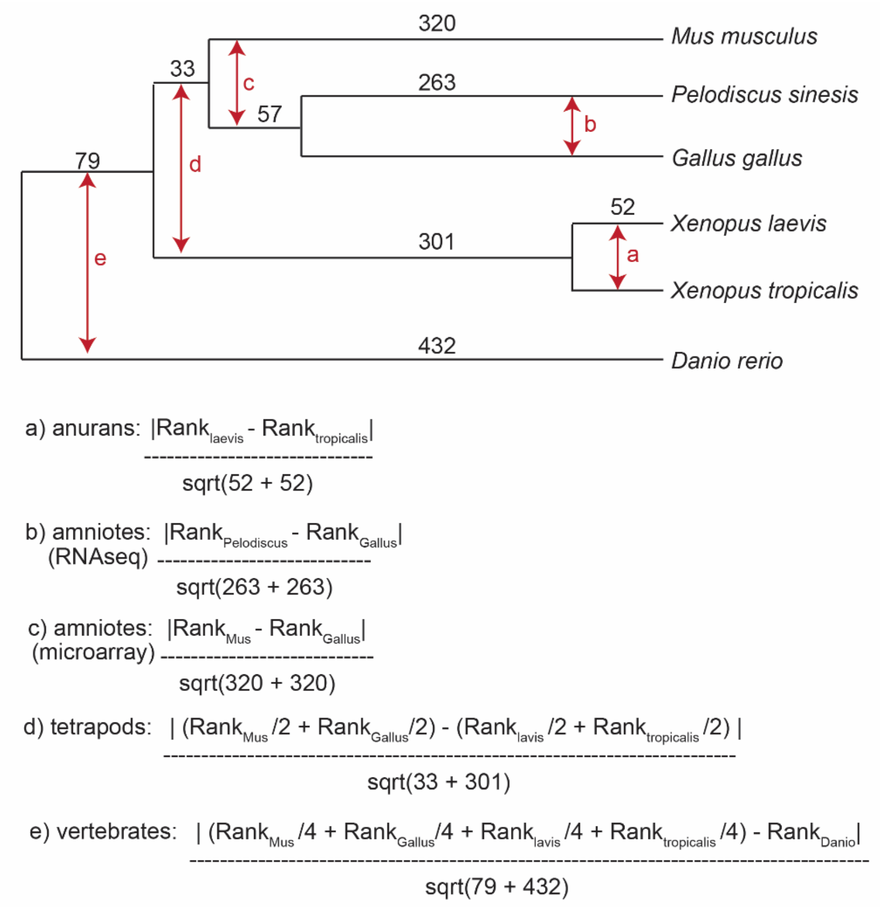

Supplementary Figure S2. To characterize expression conservation of each gene at each embryonic stage cluster at each node of the phylogeny we calculated the similarity in rank expression scaled by the divergence times of the included species.

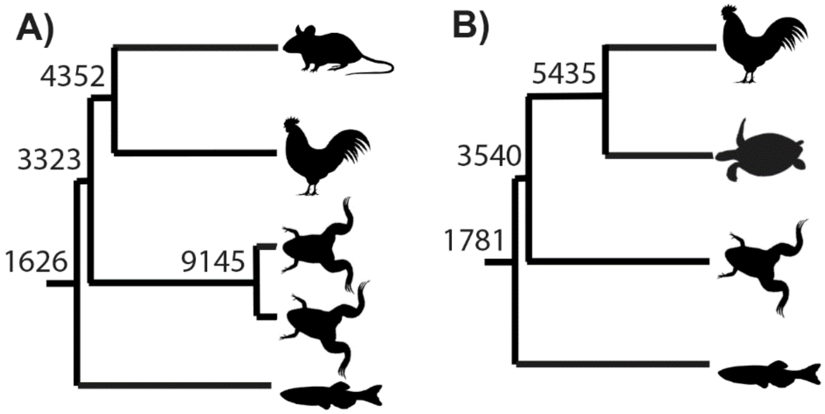

Supplementary Figure S3. The number of one-to-one orthologous identified at each node of the phylogeny for species compared using microarray data (A) and RNA-seq data (B).

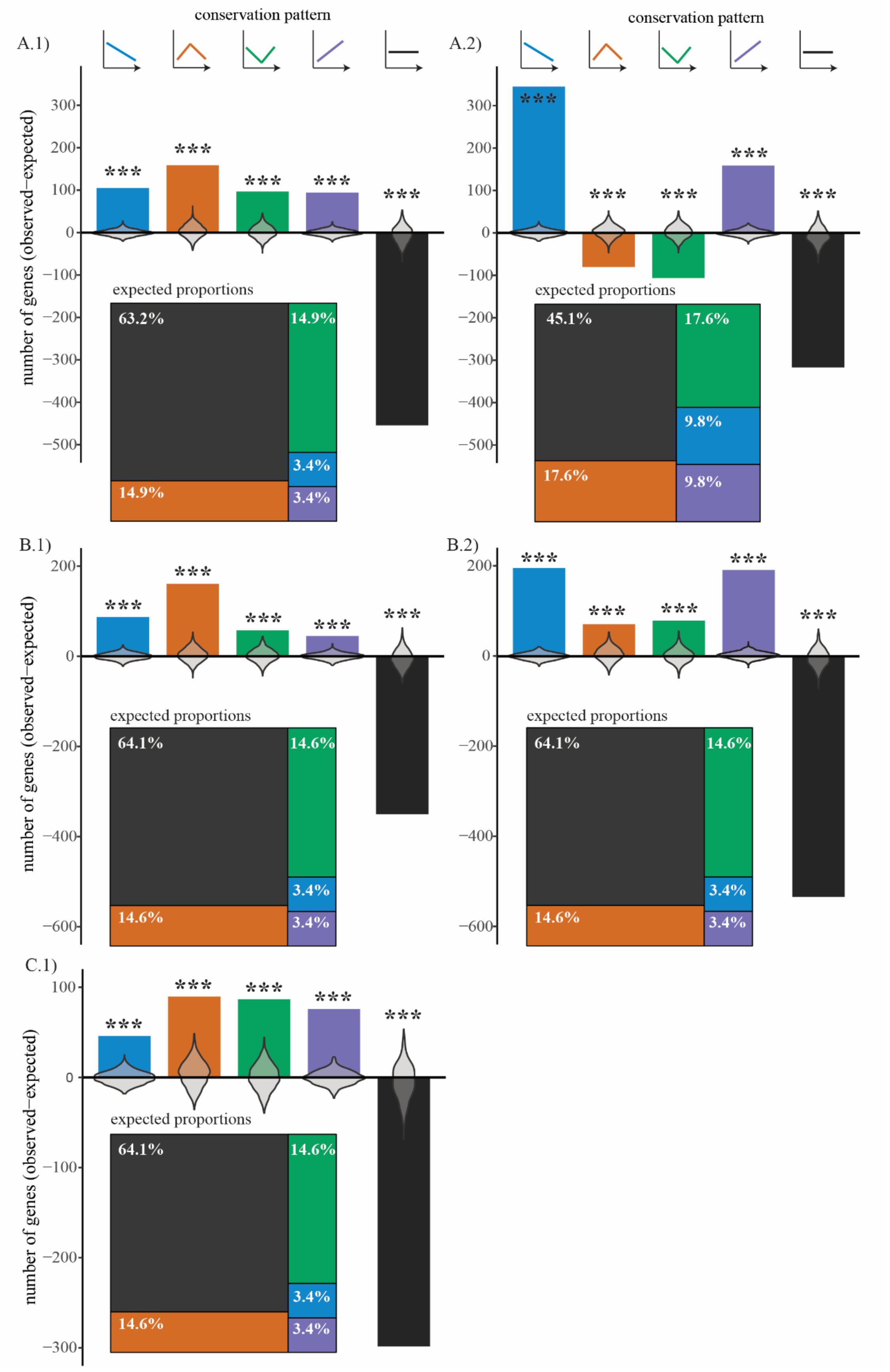
conservation pattern
A.1)
number of genes (observed−expected)
300
200
100
0
−100
−200
−300
−400
−500
***
expected proportions
63.2%
14.9%
3.4%
3.4%
14.9%
conservation pattern
A.2)
45.1%
17.6%
9.8%
9.8%
17.6%
B.1)
64.1%
14.6%
3.4%
3.4%
14.6%
B.2)
C.1)

Supplementary Figure S4. Enrichment or depletion of gene expression conservation patterns at available evolutionary nodes amniotes (A), tetrapods (B), and anurans (C) for microarray (1: left) and RNA-seq (2: right). Proportion of patterns determines the expected proportion of genes for each conservation pattern and are shown as associated treemap plots (Tennekes, 2017). The enrichment/depletion values from permutation analysis (1000 iterations) are shown as overlaying violin plots. For all plots colors indicate the expression conservation pattern. *** indicate significance at $p = 0$.
