# Supplementary material for: Comparative Transcriptomics Reveals Distinct Patterns of Gene Expression Conservation through Vertebrate Embryogenesis
Source: Genome Biol Evol. 2021 Jul 10;13(8):evab160. doi: 10.1093/gbe/evab160 (PMC8358226; doi:10.1093/gbe/evab160)
Supplement: evab160_Supplementary_Data [file evab160_supplementary_data.zip › RevisionSupportFigs.pdf]

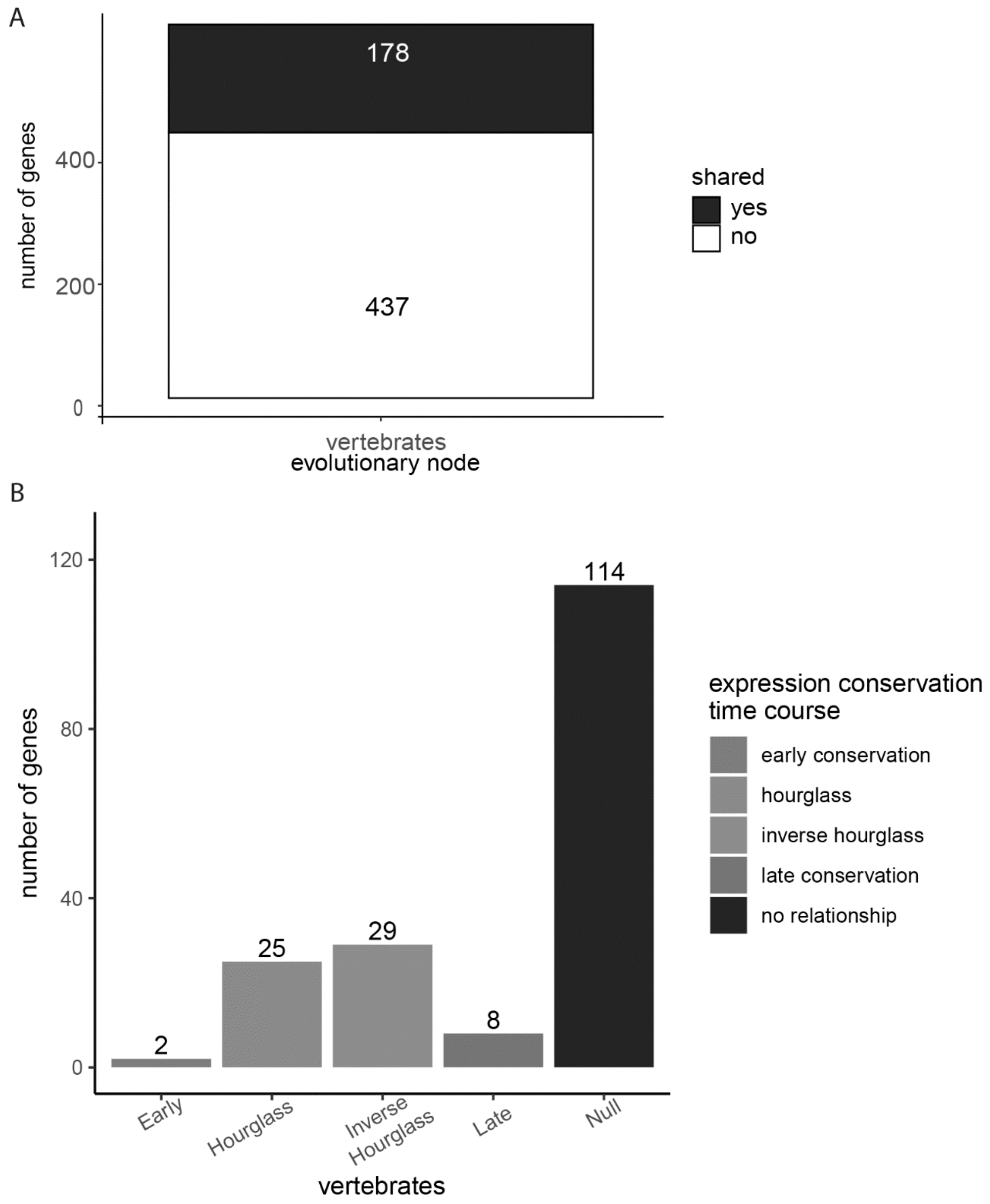

Revision Support Figure 1. Consistent Figure 8, the 615 one-to-one EggNOG orthologs shared between microarray and RNA-seq data sets largely differed in expression conservation pattern (A). Most genes with shared expression conservation patterns were “no relationship” genes (B).

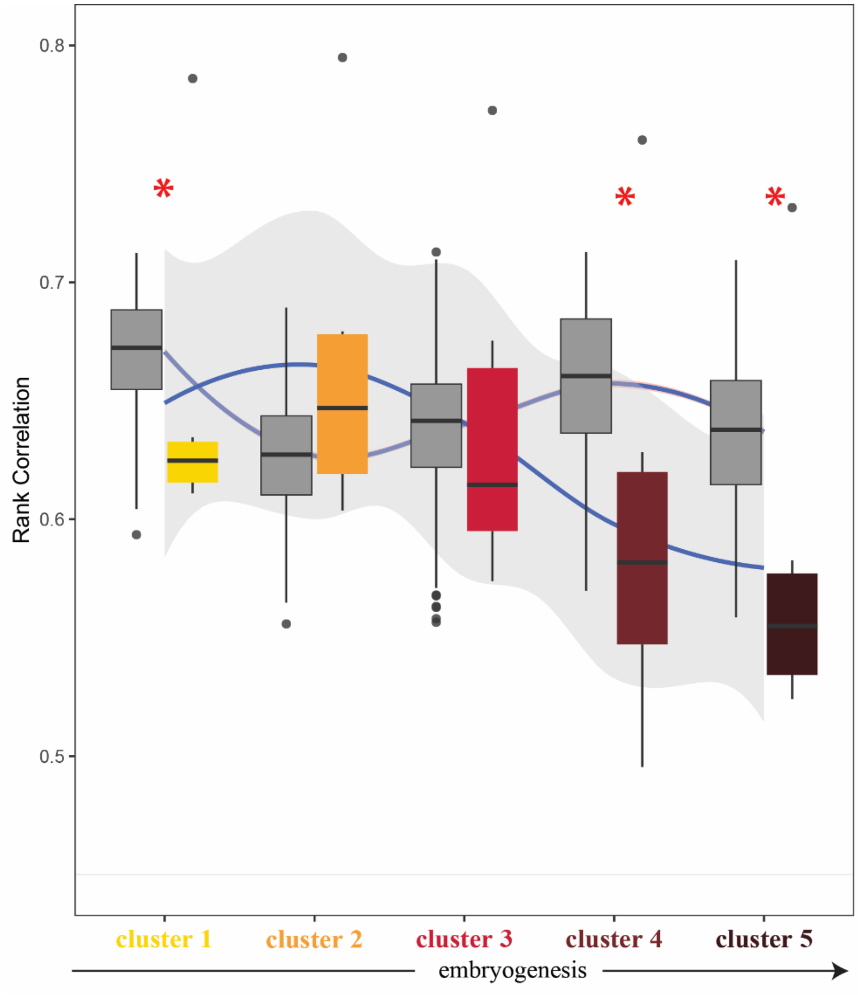

Revision Support Figure 2. Spearman rank correlations for pairwise comparisons of species at each cluster of embryogenesis for RNA-seq data after log transformation. Consistent with Figure 6B (RNA-seq without the log transformation), gene expression correlations (as a measure of conservation) vary through embryogenesis and appear to show support for a developmental hourglass. Colored boxes indicate observed correlations; grey boxes indicate rank correlations after permutation analysis randomizing stage association with cluster. Asterisks indicate that the observed median correlation differs significantly from the null expectation at $p < 0.02$.
